# Value of P300 amplitude in the diagnosis of untreated first-episode schizophrenia and psychosis risk syndrome in children and adolescents

**DOI:** 10.1186/s12888-023-05218-5

**Published:** 2023-10-12

**Authors:** Yaru Zhang, Tingyu Yang, Yuqiong He, Fanchao Meng, Kun Zhang, Xingyue Jin, Xilong Cui, Xuerong Luo

**Affiliations:** 1 Department of Psychiatry, and National Clinical Research Center for Mental Disorders, The Second Xiangya Hospital of Central South University, Changsha, Hunan 410011 China; 2 The National Clinical Research Center for Mental Disorders & Beijing Key Laboratory of Mental Disorders, Beijing Anding Hospital, Capital Medical University, Beijing, China; 3 Advanced Innovation Center for Human Brain Protection, Capital Medical University, Beijing, China

**Keywords:** Adolescents, Children, First-episode schizophrenia, P300, Psychosis risk syndrome

## Abstract

**Background:**

Identifying the characteristic neurobiological changes of early psychosis is helpful for early clinical diagnosis. However, previous studies on the brain electrophysiology of children and adolescents with psychosis are rare.

**Methods:**

This study compared P300 amplitude at multiple electrodes between children and adolescents with first-episode schizophrenia (FES, n = 48), children and adolescents with psychosis risk syndrome (PRS, n = 24), and healthy controls (HC, n = 30). Receiver operating characteristic (ROC) analysis was used to test the ability of P300 amplitude to distinguish FES, PRS and HC individuals.

**Results:**

The P300 amplitude in the FES group were significantly lower than those in the HC at the Cz, Pz, and Oz electrodes. The P300 amplitude was also significantly lower in the prodromal group than in the HC at the Pz and Oz electrodes. ROC curve analysis showed that at the Pz electrode, the P300 amplitude evoked by the target and standard stimulus showed high sensitivity, specificity, accuracy, and area under the curve value for distinguishing FES from HC individuals.

**Conclusions:**

This study found early visual P300 deficits in children and adolescents with FES and PRS, with the exclusion of possible influence of medication and chronic medical conditions, suggesting the value of P300 amplitude for the identification of early psychosis.

**Supplementary Information:**

The online version contains supplementary material available at 10.1186/s12888-023-05218-5.

## Introduction

Schizophrenia is a serious mental disease. Clinical studies have shown that the mean age at the first hospital visit or first admission of patients with schizophrenia is between 25 and 35 years [1, 2]. The etiology of the disease remains unclear, and this illness is usually accompanied by perception, thinking, and emotion impairments, reduced motivation, and neurobiological changes [3]. Schizophrenia imposes a huge burden on the patients, their families, and society due to its early age of onset, low remission rates, and high disability rates. Clinically, childhood- and adolescence-onset schizophrenia is often characterized by atypical or even highly implicit cognitive impairment, personality changes, and uncoordinated emotional and behavioral problems [4]. The prognosis of these patients is often poorer than that of adults [5], and early identification and intervention can improve the prognosis. Identifying the specific neurobiological changes of schizophrenia in the early stages may facilitate early clinical identification due to its insidious onset and atypical symptoms.

The prodromal phase of psychosis is characterized by attenuated psychotic symptoms and considerable deterioration of psychosocial functioning, and this is a critical stage due to its proneness to developing into schizophrenia [6, 7]. The prodromal phase of psychosis is usually referred to as the psychosis risk syndrome (PRS) [8], which is also known as “clinically high risk” and “ultra-high risk” [9]. A systematic review found that among children and adolescents with PRS, 17%-20% developed psychosis in one year of follow-up, whereas 23% developed psychosis in six years of follow-up, and 40% remained in the prodromal phase [10]; this has limited the justification for early use of antipsychotics. New evidence suggested that early identification and targeted treatment of patients with PRS can reduce the risk of developing psychosis [11]. Recent studies have considered using neurobiomarkers to predict PRS, provide early targeted intervention, and help elucidate the pathogenesis of schizophrenia [12, 13].

Event-related potential (ERP) is a special component of electroencephalogram evoked by a certain stimulus. Changes in ERP reflect the cognitive activities in a certain mental state. P300 is an event-related positive potential recorded on the scalp, and the P300 amplitude is usually evoked by the Oddball paradigm. The highest value of P300 is usually recorded from the central part of the scalp and the parietal lobe and is usually 300–1000 ms after stimulation [14]. At present, the mainstream view is that the P300 amplitude reflects attention resource allocation [15], sustained attention [16], staged attention shift [17, 18], and working memory update of the stimulus [19–23].

Deficits of P300 have been considered as an endophenotype of schizophrenia and used to measure neurobiological vulnerability in schizophrenia [24]. Previous studies have found reduced auditory and visual P300 amplitude in patients with first-episode schizophrenia (FES) [25–30]. Reduced auditory and visual P300 amplitude was also reported in individuals with PRS [28, 31, 32], suggesting that P300 abnormalities may occur before the full onset of psychosis. Longitudinal studies have found that after treatment in patients with schizophrenia, despite significant improvement in clinical symptoms, the decrease in auditory P300 amplitude is stable [33, 34], while the visual P300 amplitude increases with clinical improvement [35], so it is suggested that auditory P300 deficits appear to be more sensitive to schizophrenic traits, and visual P300 deficits may be more sensitive to the clinical state of schizophrenia [36, 37]. A study on P300 among individuals with PRS (aged 12.0-26.6 years) showed that after adjustment for the effect of normal age-related brain maturity on P300, younger people with PRS were more likely to have lower P300 amplitudes compared to older people with PRS, suggesting that pathological changes in P300 may be more pronounced in younger people with PRS before the typical age/maturity-related decline in P300 amplitude(P300 amplitudes increase in early development and decrease with further aging after puberty [38]). Therefore, the study of younger patients with psychosis may help to exclude the influence of changes in age/maturity-related visual P300 amplitude on early identification of neuroelectrophysiological changes in psychosis.

The diagnosis of a mental illness relies on the patient’s presentations; however, the consistency of diagnoses can be affected by individual differences, situations (e.g., time of onset and duration of the disease), information obtained, and observation of clinicians [39, 40]. Furthermore, some algorithms have been developed based on clinical and cognitive data to predict future development to schizophrenia among individuals with PRS, but their accuracy is still limited [41–43]. Therefore, recent studies have focused on identifying electrophysiological biomarkers of PRS that are associated with future development into schizophrenia among individuals with PRS [11, 44, 45].

Although P300 may play an important role in the formulation of algorithms to facilitate clinical staging of psychotic disorders [12], the extent to which visual P300 can distinguish between FES and PRS, FES and HC, and PRS and HC still needs to be further examined. Moreover, although some studies on P300 involved children and adolescents with FES and PRS, there is still a lack of relevant studies targeting this population; besides, most of the existing studies could not rule out the effect of medications. Therefore, the present study aims to examine the characteristics of the P300 amplitude among untreated children and adolescents with FES and PRS and to explore the identification value of P300 amplitude on FES and PRS in this population, thereby providing a potential neuroelectrophysiological basis for further explanation of the mechanism of early-onset schizophrenia.

## Methods

### Participants

Children and adolescents with FES (n = 48) and PRS (n = 24) aged 10–17 were recruited from psychiatric outpatient clinics and wards from August 2018 to April 2021, and HC (n = 30) were recruited through online advertisement during the same period. Patients with FES were diagnosed by two child psychiatrists according to the Diagnostic and Statistical Manual of Mental Disorders (5th Edition) [46]. Patients with PRS were diagnosed by the Structured Interview Clinical Interview Diagnosis for Psychosis-risk Syndromes, and their conditions included attenuated psychotic symptoms, brief limited intermittent psychotic episode, genetic risk, and deterioration syndrome [47, 48]. Positive and Negative Syndrome Scale (PANSS) [49] was used to assess the severity of psychiatric symptoms. Exclusion criteria included comorbidities of other mental and neurological disorders, severe physical illness, and other conditions affecting the participants’ cooperation. Healthy children and adolescents with no mental illness, no neurological disease or other serious physical disease, and no family history of mental disorders were included, based on the interview by two child psychiatrists according to Kiddie Schedule for Affective Disorders and Schizophrenia: Present and Lifetime Version (K-SADS-PL) [50]. All the subjects were right-handed, had normal visual acuity or corrected visual acuity, and had and IQ of ≥ 70 according to Wechsler Child [51]/Adult Intelligence Scale [52]. None of the subjects received any antipsychotic medication before the above evaluation. This clinical study was conducted in accordance with the latest version of the Declaration of Helsinki and was strictly reviewed and approved by the Medical Ethics Committee of The Second Xiangya Hospital of Central South University. All the subjects and their guardians have provided informed consent for the study.

### P300 paradigms

This experiment used the double-choose visual Oddball experimental paradigm. E-Prime 2.0 software (version 2.0, pstnet.com/products/e-prime/) was used to display, stimulate, and record data. Two random letters in the middle of the computer screen were the standard stimuli “W”, which appeared frequently (80%), and the target stimuli “M” appeared less frequently (20%). The experiment was divided into three blocks: one practice block and two formal blocks with a total of 265 trials. The first block was the practice block, including 15 trials; only participants with an accuracy rate of 95% or above could enter the formal experiment blocks. Each of the formal blocks was composed of 25 “M”s and 100 “W”s. Subjects were given sufficient time for a break between blocks. A random blank screen of 500–1500 ms was presented first, followed by a fixation point “+” in the middle of the screen to remind the subjects to pay attention, which lasted for 500 ms; this is followed by the “M” or “W” stimuli, which lasted for 2000 ms. Then, a random blank screen of 500–1500 ms appeared again, prompting the completion of the test. The participants were asked to respond to standard stimuli “W” and target stimuli “M” with an F/J button.

### EEG acquisition and preprocessing

#### Data acquisition

The experiment was conducted in a soundproof EEG room with appropriate temperature, with participants sitting in front of a computer monitor. German Brain Products 64-channel EEG equipment was used to record and collect the EEG data in task state, and the electrodes were placed in accordance with the position of the international 10–20 extended electrode system. The resistance of all electrodes was reduced to less than 5 KΩ. The electrode for collecting the vertical eye movement was fixed at 1 cm below the right eye. Reference electrodes were placed on the Cz electrode, and the sampling rate was 5000 Hz.

#### Preprocessing and ERP recording

MATLAB 2013b (MathWorks, Natick, MA, United States) was used for the analysis of EEG data, and EEGLAB (http://sccn.ucsd.edu/eeglab/) tool kit was used for EEG data preprocessing. The test data were imported in batches. The sampling rate was reduced to 500 Hz. Bandpass filtering was performed from 0.1 to 30 Hz, and notch filtering was performed from 48 to 52 Hz. After eliminating false responses, the test was segmented at 500 ms before the presentation of the stimuli and at 800 ms after the stimulation, and baseline correction was made at 200 ms before the presentation of stimuli. The unusable segments of the EEG data were manually eliminated, and the noisy electrodes were interpolated. Independent component analysis was used to eliminate interferences such as blinking, head movement, and power frequency interference. TP9/TP10 in the posterior mastoid process of both sides were used as reference electrodes. Segments with amplitude outside the range of -100µV to + 100µV were removed. The piecewise data of the two stimuli were superimposed and averaged to obtain the ERP waveform. The amplitude of P300 wave was defined as the average wave amplitude between 400 and 500 ms. The midline electrodes (Fz, Cz, Pz, Oz) with the most significant P300 amplitude were selected for the study.

### Statistical analysis

Statistical analysis was performed using SPSS 22.0 (IBM, Chicago, IL, United States). Data were presented using mean ± standard deviation and median (third quartile, first quartile). Inter-group comparison of gender was performed using Pearson Chi-square test, and other demographic data that did not conform to normal distribution were analyzed using Kruskal-Wallis rank sum test. As age was not matched among the three groups, it was controlled as a covariate in the subsequent analyses. The Kruskal-Wallis rank sum test was used to examine the inter-group differences in P300 amplitude evoked by the target and the standard stimulus at four electrodes (Fz, Cz, Pz, and Oz). Bonferroni correction was adopted for factors with more than two levels. The ROC function in pROC package [53] (R version 4.1.1) was used to test the ability of P300 amplitude to distinguish between FES, PRS, and HC. Accordingly, the corresponding receiver operating characteristic (ROC) curve, sensitivity, specificity, accuracy, cut-off value (cut-off value was used to distinguish between two groups of subjects, and the best cut-off value was selected using Youden’s J statistic [54]), and area under the curve (AUC) were obtained for all the groups. Benjamini & Hochberg method was used for the correction of post-hoc multiple comparisons.

## Results

### Demographic and clinical data

The Kruskal-Wallis rank sum test showed significant difference in the mean age between the FES, PRS, and HC groups (15.061 ± 1.695 vs. 13.951 ± 1.973 vs. 12.360 ± 2.357 years, respectively, *P* < 0.001). Pearson Chi-square test showed no significant difference in gender between the three groups (*P* = 0.06), with the male/female ratios being 19/29, 11/13, and 14/16, respectively. The duration of FES and PRS was 4 (7.75–2.25) and 4 (8.00–2.00) months, respectively. Demographic characteristics and PANSS scores of the FES, PRS, and HC groups are shown in Table 1.

**Table 1:** General demographic data and clinical scales of the groups of FES, PRS, and HC.

	FES (n = 48)	PRS (n = 24)	HC (n = 30)	*P*
Age (years)	15.061 ± 1.695	13.951 ± 1.973	12.360 ± 2.357	< 0.001
Gender (male/female)	19/29	11/13	14/16	0.060
Duration of disease (months)	4 (7.75, 2.25)	4 (8.00, 2.00)		
PANSS score				
Positive symptoms	20.05 ± 5.14	15.13 ± 4.24		
Negative symptoms	20.26 ± 6.93	16.60 ± 5.61		
General psychopathological symptoms	41.58 ± 8.90	27.87 ± 6.58		

Notes: Data were presented by mean ± standard deviation and median (third quartile, first quartile). n: the number of patients

Abbreviations: FES, first-episode schizophrenia; PRS, psychosis risk syndrome; HC, healthy controls; PANSS, Positive and Negative Syndrome Scale

### P300 amplitude

**Fig. 1:**
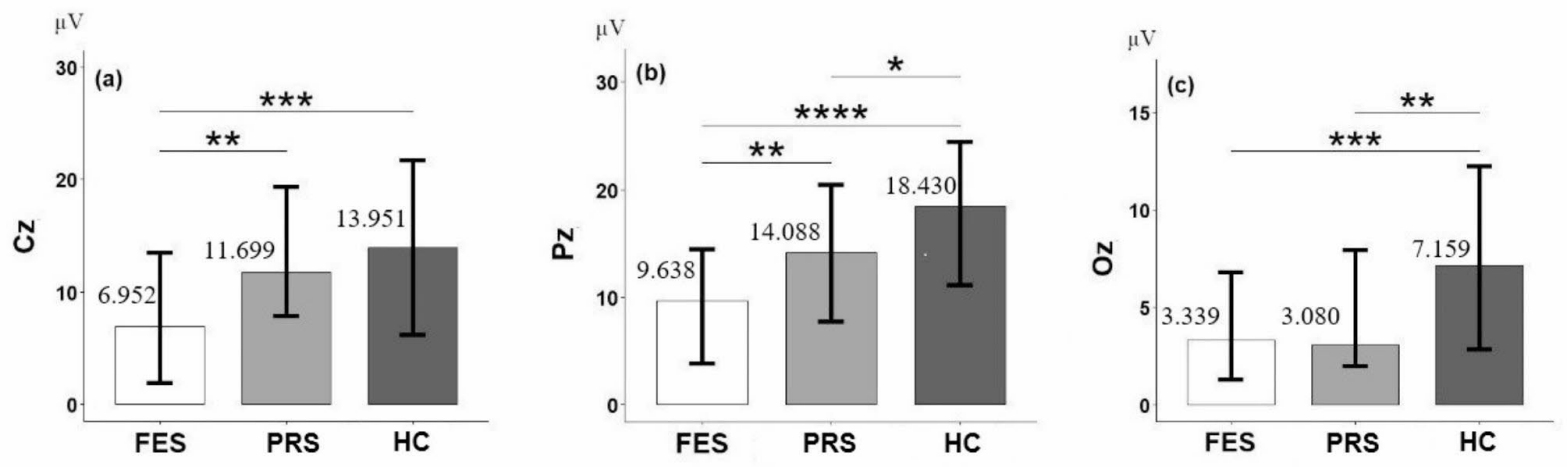
Note: Group differences in P300 amplitude evoked by target stimulus at different electrodes. (**a**) Cz electrode, (**b**) Pz electrode, (**c**) Oz electrode. *: *p* < 0.05; **<*p* < 0.01; ***<*p* < 0.001; ****<*p* < 0.0001. FES, first-episode schizophrenia; PRS, psychosis risk syndrome; HC, healthy controls

The comparison of P300 amplitude evoked by the target stimulus showed the following results: at the Fz electrode, no significant difference was found between groups (*P* > 0.05); at the Cz electrode, the P300 amplitude was significantly different between groups (H = 16.49, *P* < 0.001). Post-hoc test showed that the P300 amplitude of the FES group (6.952 ± 6.088 µV) was significantly lower than that of the PRS group (11.699 ± 7.449 µV) and HC (13.951 ± 7.759 µV), but no significant difference was found between the PRS group and HC (*P* = 0.36), as shown in Fig. 1(a). At the Pz electrode, the P300 amplitude was significantly different between groups (H = 26.37, *P* < 0.0001). Post-hoc test showed that the amplitude of the FES group (9.638 ± 6.361 µV) was significantly lower than that of the PRS group (14.088 ± 6.380 µV) and HC (18.430 ± 7.475 µV), and the amplitude of the PRS group was also significantly lower than that of HC (*P* = 0.035), as shown in Fig. 1(b). At the Oz electrode, the P300 amplitude showed significant difference between groups (H = 14.21, *P* < 0.001). Post-hoc test showed that the amplitude of the FES group (3.339 ± 3.538 µV) and PRS group (3.080 ± 4.354 µV) was significantly lower than that of HC (7.159 ± 5.109 µV), but no significant difference was found between the FES group and the PRS group (*P* = 0.75), as shown in Fig. 1(c).

**Fig. 2:**
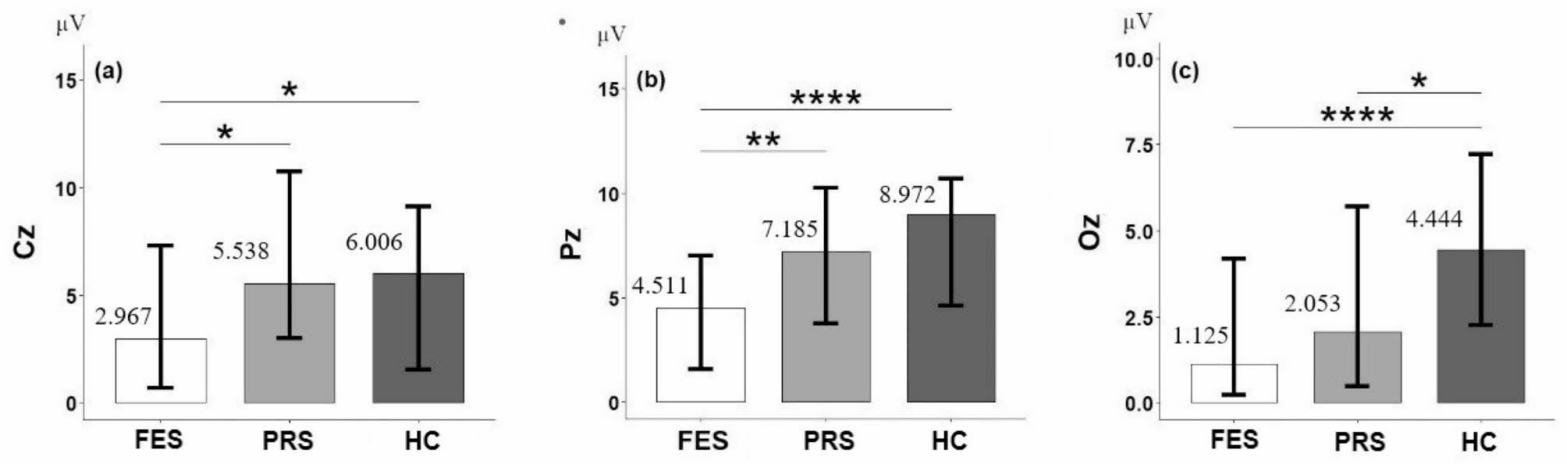
Note: Group differences in P300 amplitude evoked by standard stimulus at different electrodes. (**a**) Cz electrode, (**b**) Pz electrode, (**c**) Oz electrode. *: *p* < 0.05; **<*p* < 0.01; ***<*p* < 0.001; ****<*p* < 0.0001. FES, first-episode schizophrenia; PRS, psychosis risk syndrome; HC, healthy controls

The comparison of P300 amplitude evoked by normal stimulus showed the following results: at the Fz electrode, no significant difference was found between groups (*P* > 0.05). At the Cz electrode, the P300 amplitude was significantly different between groups (H = 8.12, *P* = 0.017). Post-hoc test showed that the amplitude of the FES group (2.967 ± 3.717 µV) was significantly lower than that of the PRS group (5.538 ± 4.712 µV) and HC (6.006 ± 5.533 µV), and no significant difference was found between the PRS group and HC (*P* = 0.90), as shown in Fig. 2(a). At the Pz electrode, the amplitude of P300 was significantly different between groups (H = 24.40, *P* < 0.0001). Post-hoc test showed that the amplitude of the FES group (4.511 ± 3.850 µV) was significantly lower than that of the PRS group (7.185 ± 4.556 µV) and HC (8.972 ± 5.046 µV), but no significant difference was found between the PRS group and HC (*P* = 0.20), as shown in Fig. 2(b). At the Oz electrode, the P300 amplitude was significantly different between groups (H = 18.49, *P* < 0.0001). Post-hoc test showed that the amplitude of the FES group (1.125 ± 2.653 µV) and PRS group (2.053 ± 2.902 µV) was significantly lower than that of HC (4.444 ± 3.434 µV), but no significant difference was found between the FES group and the PRS group (*P* = 0.41), as shown in Fig. 2(c).

Supplementary Figure A.1, Figure A.2, and Figure A.3 showed the ERP grand averages evoked by the standard and target stimuli at different electrodes (Cz, Pz, and Oz respectively) for all the three groups. Supplementary figure B showed the topographic map of P300 for each group.

### ROC analysis

**Fig. 3:**
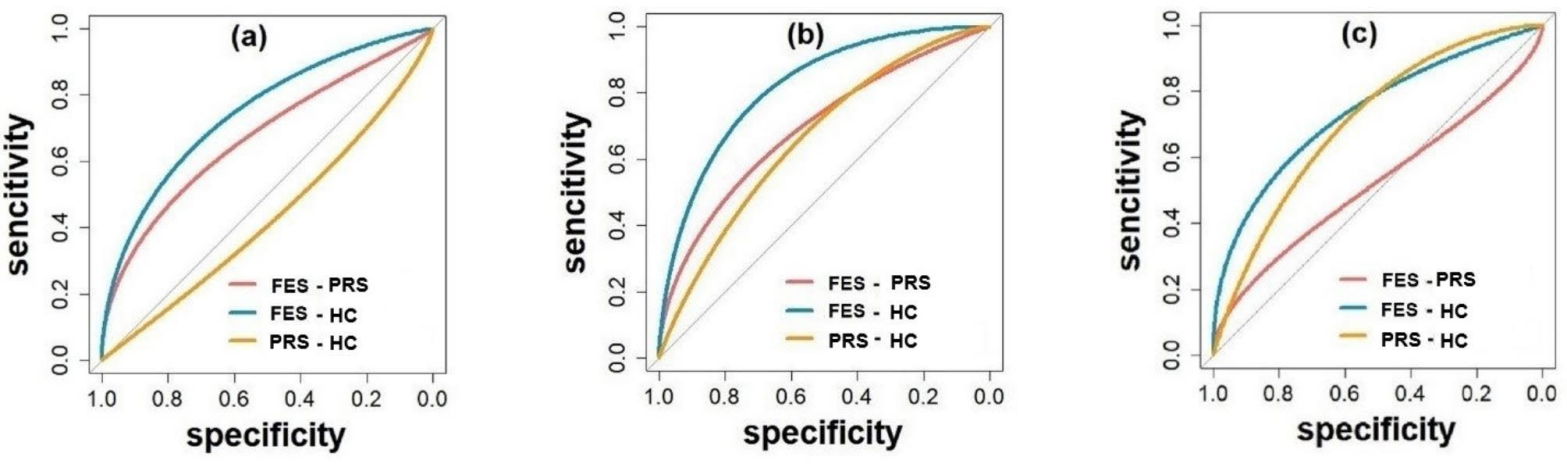
Note: ROC curves of P300 amplitude evoked by target stimulus at different electrodes. Slashes indicate random levels. (**a**) Cz electrode, (**b**) Pz electrode, (**c**) Oz electrode. FES, first-episode schizophrenia; PRS, psychosis risk syndrome; HC, healthy controls

**Fig. 4:**
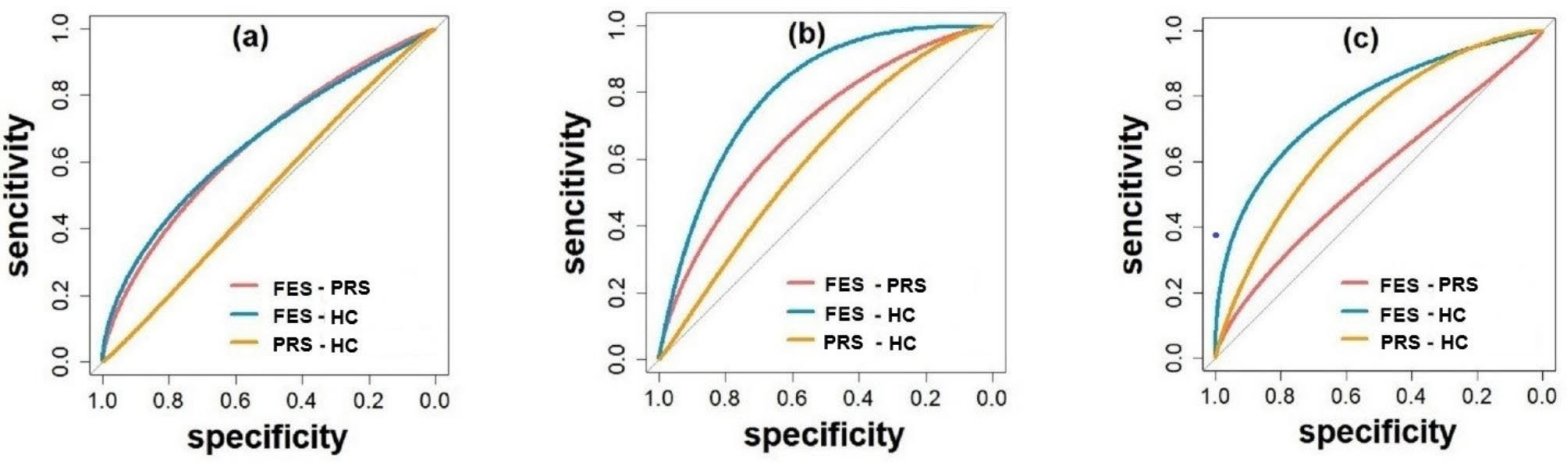
Note: ROC curves of P300 amplitude evoked by standard stimulus at different electrodes. Slashes indicate random levels. (**a**) Cz electrode, (**b**) Pz electrode, (**c**) Oz electrode. FES, first-episode schizophrenia; PRS, psychosis risk syndrome; HC, healthy controls

At the Pz electrode, the P300 amplitude evoked by the target stimulus and standard stimulus showed high sensitivity (0.97, 0.73), specificity (0.56, 0.83), accuracy (0.72, 0.79), and AUC value (0.83, 0.82) for distinguishing FES from HC. All the results are presented in Tables 2 and 3; Fig. 3, and Fig. 4.

**Table 2:** Sensitivity, specificity, accuracy, cut-off value, and AUC value corresponding to ROC curve (P300 amplitude evoked by target stimulus)

Characteristic components	Group-Group	Sensitivity	Specificity	Accuracy	Cut-off value	AUC value
Cz	FES-PRS	0.625	0.8125	0.75	11.27083	0.70
	FES-HC	0.66	0.79	0.74	10.69075	0.75
	PRS-HC	0.5	0.54	0.52	12.74969	0.43
Pz	FES-PRS	0.625	0.83	0.76	15.01199	0.71
	FES-HC	0.97	0.56	0.72	9.125635	0.83
	PRS-HC	0.33	1	0.63	22.69045	0.67
Oz	FES-PRS	0.33	0.875	0.69	0.84	0.52
	FES-HC	0.73	0.73	0.73	4.572459	0.75
	PRS-HC	0.73	0.67	0.70	4.563805	0.72

Abbreviations: Cz, Cz electrode; Pz, Pz electrode; Oz, Oz electrode; AUC, area under the curve; FES, first-episode schizophrenia; PRS, psychosis risk syndrome; HC, healthy controls

**Table 3:** Sensitivity, specificity, accuracy, cut-off value, and AUC value corresponding to ROC curve (P300 amplitude evoked by standard stimulus)

Characteristic components	Group-Group	Sensitivity	Specificity	Accuracy	Cut-off value	AUC value
Cz	FES-PRS	0.33	0.875	0.69	0.8405736	0.52
	FES-HC	0.73	0.73	0.73	4.572459	0.75
	PRS-HC	0.73	0.67	0.70	4.563805	0.72
Pz	FES-PRS	0.67	0.81	0.76	5.79646	0.72
	FES-HC	0.73	0.83	0.79	6.11725	0.82
	PRS-HC	0.6	0.625	0.61	7.47215	0.60
Oz	FES-PRS	0.29	0.875	0.68	3.331363	0.56
	FES-HC	0.73	0.8125	0.78	2.656331	0.79
	PRS-HC	0.73	0.71	0.72	2.619938	0.71

Abbreviations: Cz, Cz electrode; Pz, Pz electrode; Oz, Oz electrode; AUC, area under the curve; FES, first-episode schizophrenia; PRS, psychosis risk syndrome; HC, healthy controls

## Discussion

In the present study, we found that the P300 amplitude in the FES group was significantly lower than that in the HC group, which was consistent with prior studies on patients with schizophrenia [55–59]. The P300 amplitude reflects the attention resources of the subjects, with a higher amplitude indicating a higher attention level of the individual [15]. Cognitive attention resource allocation and executive function are affected by functionally or anatomically different brain networks involving a variety of brain regions, such as the parietal cortex and cingulate gyrus [60]. The general reduction in P300 amplitude may indicate impaired attention-driven working memory as reflected by different responses to the target and the standard stimuli during the oddball task in patients with FES [11, 61]. A reduced P300 amplitude may reflect other deficiency in complex cognitive processes, including working memory and contextual updating [62–64]. Strandburg et al. [65] reported that reduced P300 amplitude might indicate uncertainty to the target stimulus among people with FES. In a visual recognition task, P300 amplitude was significantly lower in patients with schizophrenia compared to HC, indicating that patients with schizophrenia might have difficulty integrating incoming information based on past experience [66]. Furthermore, there are few studies reporting the neuropsychological relevance of P300 in schizophrenia. In patients with schizophrenia, P300 amplitude was found associated with poor verbal memory and learning performance [67, 68]. Some studies also reported a negative association between P300 amplitude and the severity of negative symptoms of schizophrenia [69, 70].

The present study found that the P300 amplitude decreased most significantly in the parietal lobe of patients with FES, which was consistent with most prior findings [71, 72]. The widespread cognitive deficits in schizophrenia are largely due to the inability to regulate activity flexibly in the posterior parietal cortex, which stores information in the current working memory [73]. A study of childhood schizophrenia found that gray matter abnormalities originated in the parietal lobes, these deficits progressed anteriorly into frontal lobes, suggesting that structural abnormalities in the parietal lobes may appear early in the disease [74].

We found a decrease in P300 amplitude in patients with PRS compared with HC, which was in line with previous findings [75–80]. A systematic review concluded that reduced P300 amplitude is a reliable finding among individuals with PRS and therefore has the potential to be an indicator for higher risk of psychosis [11]. However, few studies have compared P300 amplitudes between people with PRS that developed into schizophrenia and those who do not, and the results are inconclusive [13, 78, 81]. A recent study showed that individuals with PRS who achieved remission during a follow-up period of at least 22 months had a baseline P300 amplitude similar to that of HC and higher than that of individuals with PRS who subsequently converted to psychosis and who still had symptoms [81]. Taken together, P300 amplitudes appear to be helpful for the screening of individuals at high risk of developing psychosis, but more longitudinal studies with follow-up are needed to determine its potential for clinical use.

The present study found that the changes in P300 amplitude among individuals with PRS mainly happened in the parietal and occipital lobes. Several imaging studies have supported structural changes in the parietal and occipital lobes in patients with PRS. For example, a longitudinal whole-brain imaging study of young patients with PRS showed that ventricular dilatation in the parietal cortex was associated with a progressive reduction in gray matter, whereas no such association was seen in normal subjects [82]. Another imaging study found a significant reduction in bilateral occipital gray matter volume in individuals with a high genetic risk of psychosis [83]. Some basic symptoms may show up in childhood before the onset of psychosis [84] and may be related to subtle disturbances that affect the development of an individual’s integrated sense of self. For instance, abnormalities in the inferior parietal lobe in childhood may lead to impairments in sensory integration and the ability to distinguish between self and others. During subsequent development in adolescence, structural and functional changes in brain regions that form the neural circuits related to the ego may further contribute to abnormal processing of self-experience and self-reflection, which might leading to psychosis later in life [85].

We also found that HC exhibited the highest P300 amplitude at the Pz electrode, followed by individuals with PRS and the lowest in individuals with FES, which, to our knowledge, is the first time that a descending hierarchy of P300 amplitude has been reported in patients with psychosis. A previous study on visual P300 in patients with PRS and FES in early adulthood [26] and another study of auditory and visual P300 in patients with schizophrenia and PRS [12] found no significant difference in P300 amplitude between patients with PRS and those with schizophrenia, although a reduction in P300 amplitude was found in the two patient groups compared to HC. A study on auditory P300 in children and adolescents with schizophrenia and PRS found although P300 amplitude was reduced in individuals with schizophrenia, there was no significant differences in P300 amplitude between HC and individuals with PRS, which, according to the authors, might reflect clinical heterogeneity among people with PRS [86]. Besides, most of the subjects included in the above studies had already been on antipsychotic medications, thus, the influence of medications on the results could not be completely ruled out. The present study provides additional evidence that the reduction in visual P300 amplitude reflects ongoing pathophysiological processes from PRS to FES, making it a potential marker of vulnerability in early psychosis.

The present study suggested that the P300 amplitude is a potential indicator to distinguish between FES, PRS and HC individuals with high identification sensitivity. In the present study, the AUC of P300 amplitude evoked by target/standard stimulus at the Pz electrode was 0.83/0.82 in distinguishing FES from HC among children and adolescence, which indicated a good diagnostic value of AUC of P300 amplitude. However, the present study found that the P300 amplitude was only moderately good at distinguishing PRS from HC among children and adolescents, which might be attributed to the heterogeneity of clinical symptoms among children and adolescents with PRS as well as multiple causal biological mechanisms [7]. Given that the symptoms of some children and adolescents with PRS might be transient, mild, or insidious [10], early neurophysiological impairment was not evident. Moreover, the experimental task in the present study was relatively simple, and more difficult tasks are needed to distinguish between subtypes of the same disease. With regard to the heterogeneity of individuals and experimental paradigms, further studies are also needed to examine the value of EEG indicators as a diagnostic tool for psychosis.

The present study has some limitations; thus, the results obtained should be interpreted with caution. First, the sample size of this study was limited, especially for the PRS group; thus, these results must be confirmed in a larger-scale study. Second, this study was a cross-sectional study without the longitudinal follow-up for disease outcomes and neurophysiological dynamics. Longitudinal studies with a follow-up period for PRS may further clarify whether P300 can be used as a neurophysiological marker to predict the onset and prognosis of psychosis. Despite these limitations, this study was the first to find early visual P300 deficits in children and adolescents with FES and PRS, with the exclusion of the influence of medications and chronic medical conditions, which may provide a neuroelectrophysiological basis for the explanation of pathological mechanisms of early-onset psychosis as well as a basis for the use of P300 amplitude in the early diagnosis of psychosis.

### Electronic supplementary material

Below is the link to the electronic supplementary material.

Supplementary Material 1: The group mean ERPs, topographic maps and methodological supplement.

## Data Availability

The data that support the findings of this study are available from the corresponding author upon reasonable request.
