# Supplementary material for: Value of P300 amplitude in the diagnosis of untreated first-episode schizophrenia and psychosis risk syndrome in children and adolescents
Source: BMC Psychiatry. 2023 Oct 12;23:743. doi: 10.1186/s12888-023-05218-5 (PMC10571359; doi:10.1186/s12888-023-05218-5)
Supplement: Supplementary file 1 — Supplementary Material 1: The group mean ERPs, topographic maps and methodological supplement. [file 12888_2023_5218_MOESM1_ESM.docx]

Figure A.1 The ERP grand averages evoked by the standard and target stimuli for three groups at Cz electrode.


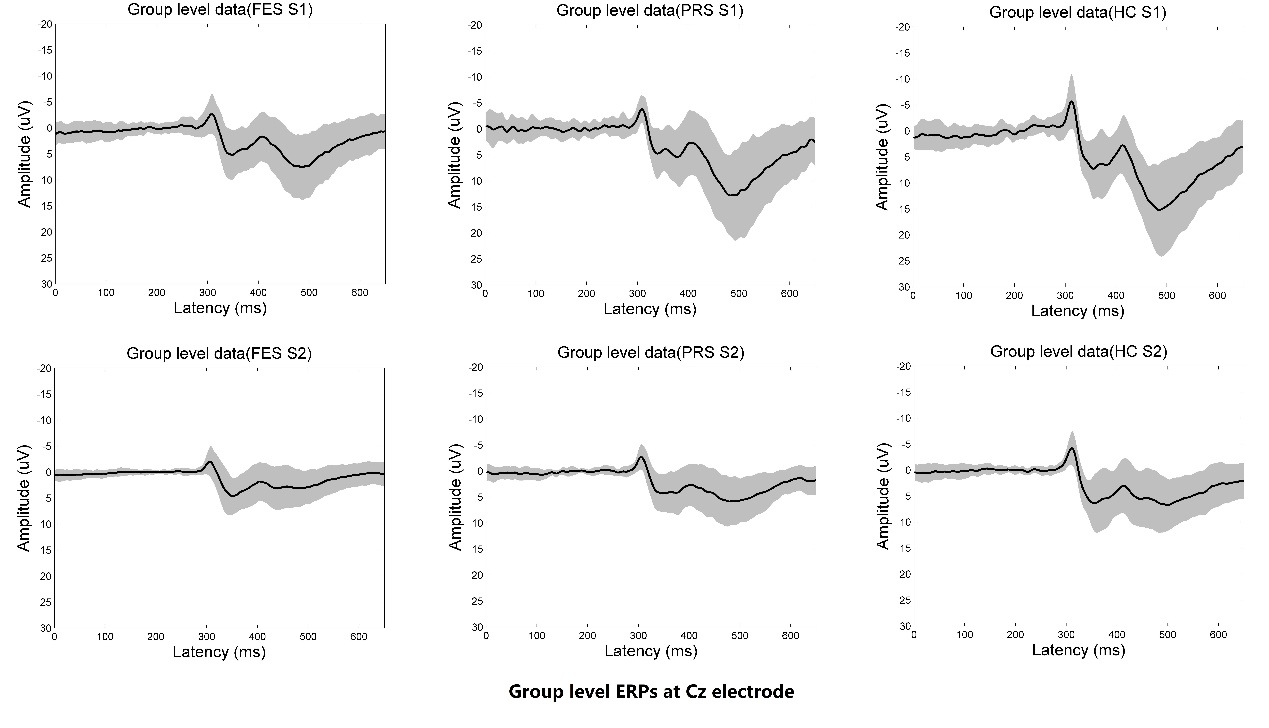


Figure A.2 The ERP grand averages evoked by the standard and target stimuli for three groups at Pz electrode.


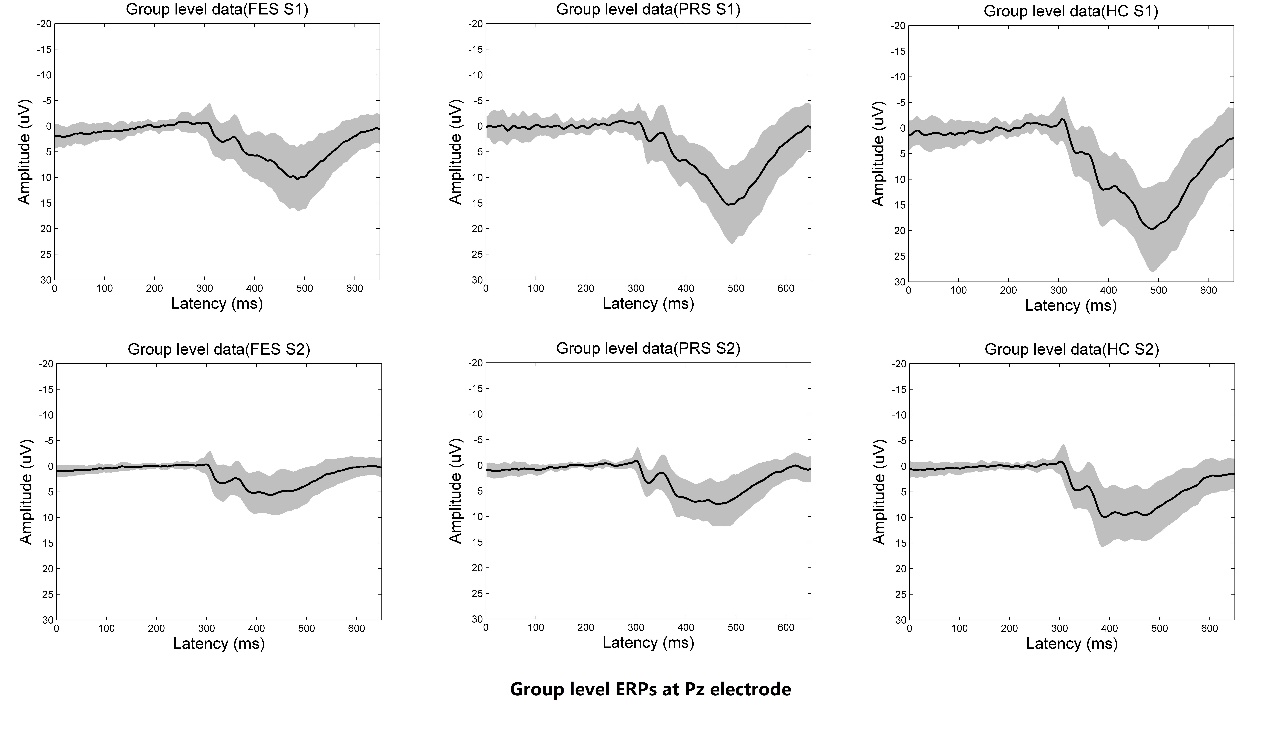


Figure A.3 The ERP grand averages evoked by the standard and target stimuli for three groups at Oz electrode.


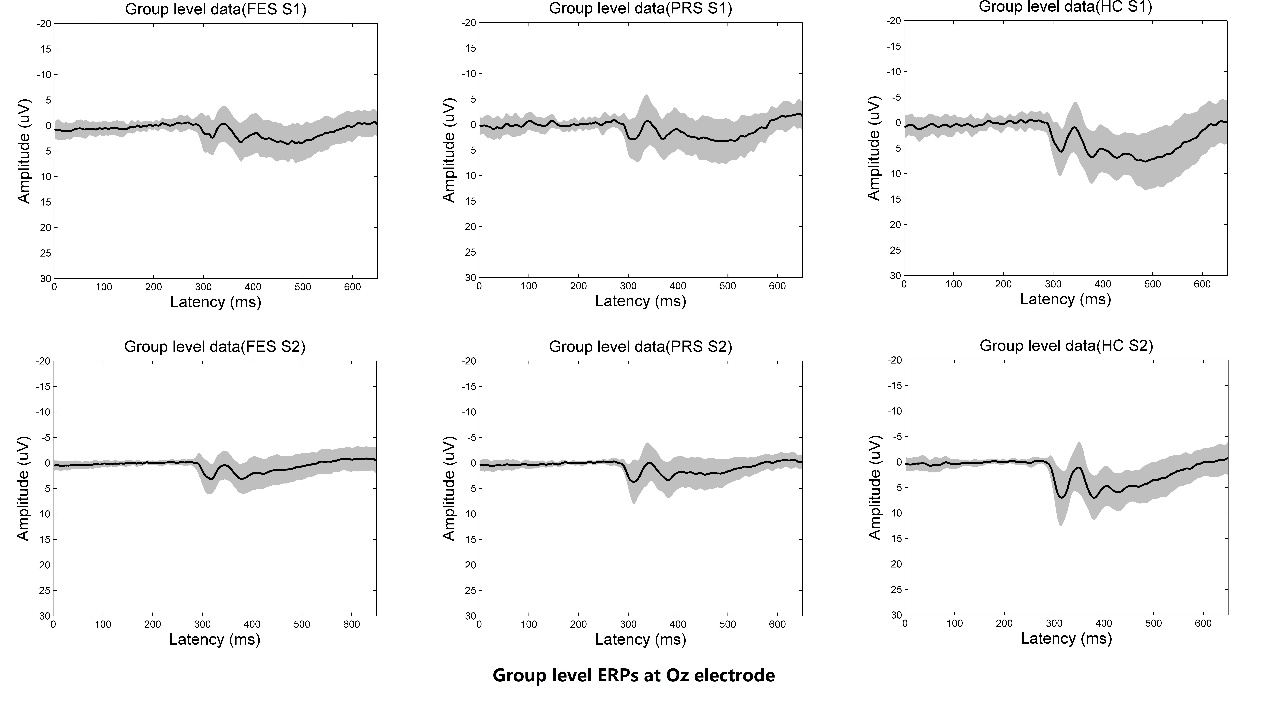


1. Grand averaged amplitudes of ERPs for first-episode schizophrenia(FES), psychosis risk syndrome (PRS) and healthy control(HC) groups at Cz/Pz/Oz electrode respectively. The black line is the mean ERPs and the gray area is the standard deviation of the ERPs.


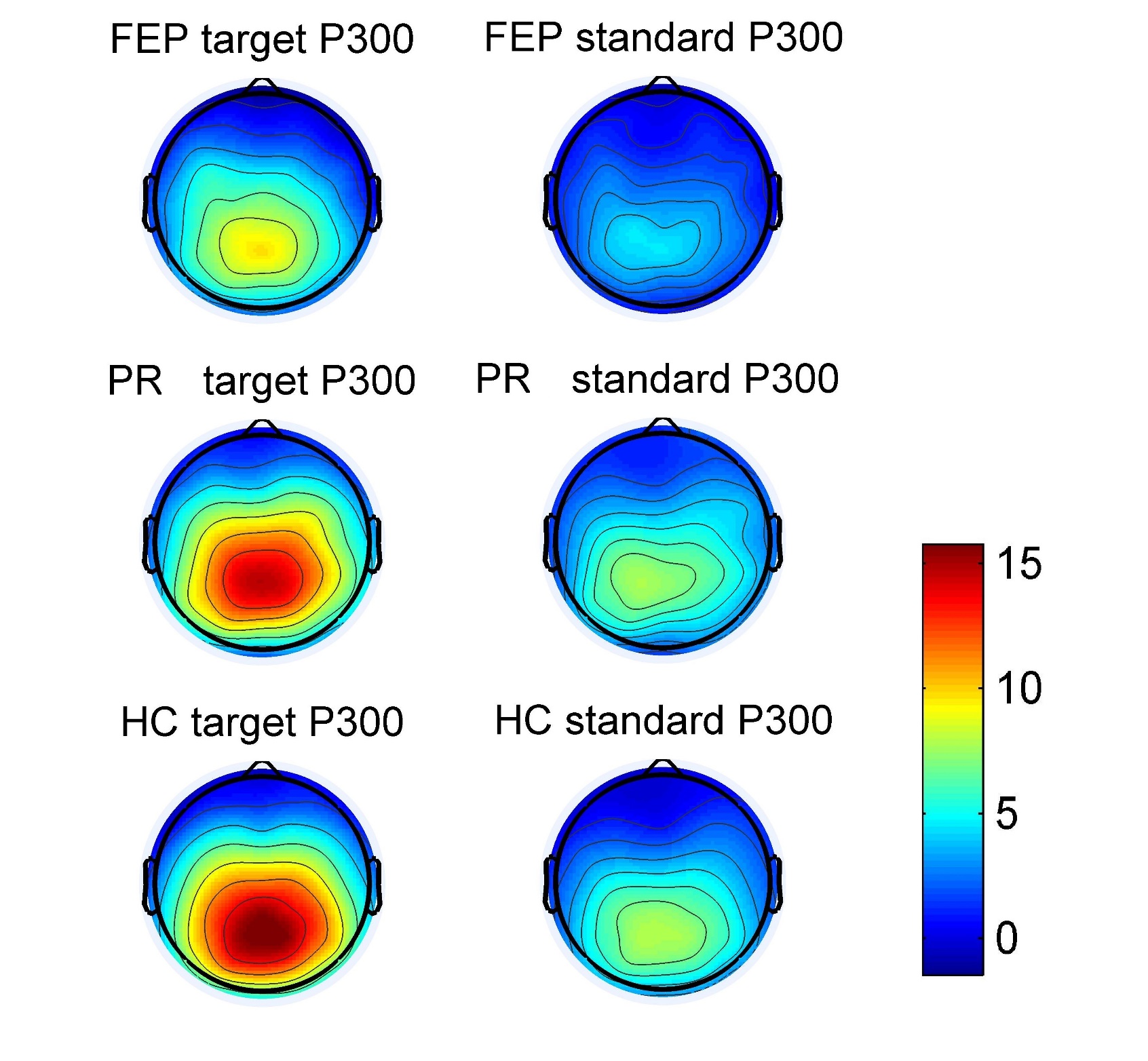
Figure B Topographic map of P300 for three groups.

1. Topographic map of P300 for first-episode (FE) schizophrenia, psychosis risk syndrome (PR) and healthy control(HC) groups. The color of the colorbar on the right, from blue to red, represents the amplitude from small to large.

Figure C The calculation method of “specificity”, “sensitivity”, “accuracy”.

| ‘tn’ | True negative count |  |
| --- | --- | --- |
| ‘tp’ | True positive count |  |
| ‘fn’ | False negative count |  |
| ‘fp’ | False positive count |  |
| ‘specificity’ | Specificity | tn / (tn+fp) |
| ‘sensitivity’ | Sensitivity | tp / (tp+fn) |
| ‘accuracy’ | Accuracy | (tp+tn) / ((tn+fp+tp+fn) |
